# Phase I dose escalation study and pilot efficacy analysis of LXI-15029, a novel mTOR dual inhibitor, in Chinese subjects with advanced malignant solid tumors

**DOI:** 10.1186/s12885-023-11578-8

**Published:** 2023-12-06

**Authors:** Jiani Wang, Lin Gui, Yuxin Mu, Jiayu Wang, Yihebali Chi, Zhenteng Liu, Qing Li, Binghe Xu

**Affiliations:** 1 Department of Medical Oncology, National Cancer Center/National Clinical Research Center for Cancer/Cancer Hospital, Chinese Academy of Medical Sciences and Peking Union Medical College, No. 17, Panjiayuannanli, Chaoyang District, Beijing, 100021 China; 2 Shandong Luoxin Pharmaceutical Group Co., Ltd., Linyi, 276017 China; 3 State Key Laboratory of Molecular Oncology, National Cancer Center/National Clinical Research Center for Cancer/Cancer Hospital, Chinese Academy of Medical Sciences and Peking Union Medical College, No. 17, Panjiayuannanli, Chaoyang District, Beijing, 100021 China

**Keywords:** LXI-15029, mTORC1/2 dual inhibitor, Metastatic solid tumors, First-in-human trial

## Abstract

**Background:**

The mammalian target of rapamycin (mTOR) kinase, a central component of the PI3K/AKT/mTOR pathway, plays a critical role in tumor biology as an attractive therapeutic target. We conducted this first-in-human study to investigate the safety, pharmacokinetics (PK), and pilot efficacy of LXI-15029, an mTORC1/2 dual inhibitor, in Chinese patients with advanced malignant solid tumors.

**Methods:**

Eligible patients with advanced, unresectable malignant solid tumors after failure of routine therapy or with no standard treatment were enrolled to receive ascending doses (10, 20, 40, 60, 80, 110, and 150 mg) of oral LXI-15029 twice daily (BID) (3 + 3 dose-escalation pattern) until disease progression or intolerable adverse events (AEs). The primary endpoints were safety and tolerability.

**Results:**

Between June 2017 and July 2021, a total of 24 patients were enrolled. LXI-15029 was well tolerated at all doses. Only one dose-limiting toxicity (grade 3 increased alanine aminotransferase) occurred in the 150 mg group, and the maximum tolerated dose was 110 mg BID. The most common treatment-related AEs were leukocytopenia (41.7%), increased alanine aminotransferase (20.8%), increased aspartate aminotransferase (20.8%), prolonged electrocardiogram QT interval (20.8%), and hypertriglyceridemia (20.8%). No other serious treatment-related AEs were reported. LXI-15029 was absorbed rapidly after oral administration. The increases in the peak concentration and the area under the curve were greater than dose proportionality over the dose range. Eight patients had stable disease. The disease control rate was 40.0% (8/20; 95% CI 21.7–60.6). In evaluable patients, the median progression-free survival was 29 days (range 29–141).

**Conclusions:**

LXI-15029 demonstrated reasonable safety and tolerability profiles and encouraging preliminary antitumor activity in Chinese patients with advanced malignant solid tumors, which warranted further validation in phase II trials.

**Trial registration:**

NCT03125746(24/04/2017),http://ClinicalTrials.gov/show/NCT03125746

## Introduction

The mammalian/mechanistic target of rapamycin (mTOR) is a pivotal regulatory protein kinase involved in multiple tumor signaling pathways and plays an important role in cell growth, proliferation, angiogenesis, protein synthesis, and cell apoptosis [1]. The overactivation of the middle-to-upstream signals in the mTOR pathway is closely related to the occurrence of tumors [1]. For example, the overexpression of phosphatidylinositol 3-kinase (PI3K) and protein kinase B (PKB), imbalance of the upstream negative regulator phosphatase and tensin homolog (PTEN), tuberous sclerosis complex 1/2 (TSC1/2), and liver kinase B1 (LKB1) can result in the overactivation of the mTOR signaling pathway, causing cell mutation and promoting a variety of malignant tumors, including prostatic cancer, breast cancer, and endometrial cancer [2–4]. The downstream factors of mTOR are also involved in the occurrence of tumors; the gene and protein overexpression of the eukaryotic initiation factor 4E (eIF4E) and ribosomal protein S6 kinase 1 (S6K1) is found in a variety of cancers [1, 5]. mTOR can also control the production of cyclin D1 and c-Myc and affect the expression of the vascular endothelial growth factor (VEGF), which are all indirectly but closely related to tumors through enhancing the expression of hypoxia-inducible factor (HIF) [6]. Therefore, mTOR has become one of the most important targets for anticancer drugs [6].

Everolimus, an mTOR inhibitor, has been approved as a single agent for the treatment of advanced renal cell carcinoma and pancreatic neuroendocrine tumors based on its anti-angiogenic and antitumor activity [7, 8]. In addition, combination therapy with everolimus and endocrine therapy (such as exemestane or letrozole) showed encouraging anticancer activity in pre- and postmenopausal patients with advanced breast cancer [9, 10]. Therefore, recent advances in research on mTOR inhibition involve various compounds developed to disrupt the metabolic process of cancer cells through the PI3K/AKT/mTOR pathway [11, 12].

In mammalian cells, mTOR is mainly assembled in two different complexes (mTOR complex 1 [mTORC1] and complex 2 [mTORC2]) that have different regulatory mechanisms involved in different tumor biological functions, such as cell proliferation, growth, and metabolism, through the activation of different downstream molecules [13, 14]. Thus, research on mTOR modulation involves various compounds to interfere with the metabolic processes in cancer cells [15]. The allosteric mTOR inhibitors (e.g., everolimus, temsirolimus, and sirolimus) approved by the Food and Drug Administration (FDA) for human cancers can inhibit mTORC1 but have no obvious inhibiting effect on mTORC2. Although rapamycin and its analogs (rapalogs) inhibit mTORC1 xenobiologically and specifically, their limitations in effective anticancer therapy have been reported [16]. The inhibition of mTORC1 alone without complete inhibition of the mTOR signaling pathway can produce the feedback activation of other signal pathways, such as AKT phosphorylation, limiting the antitumor activity [17, 18].

**Fig. 1:**
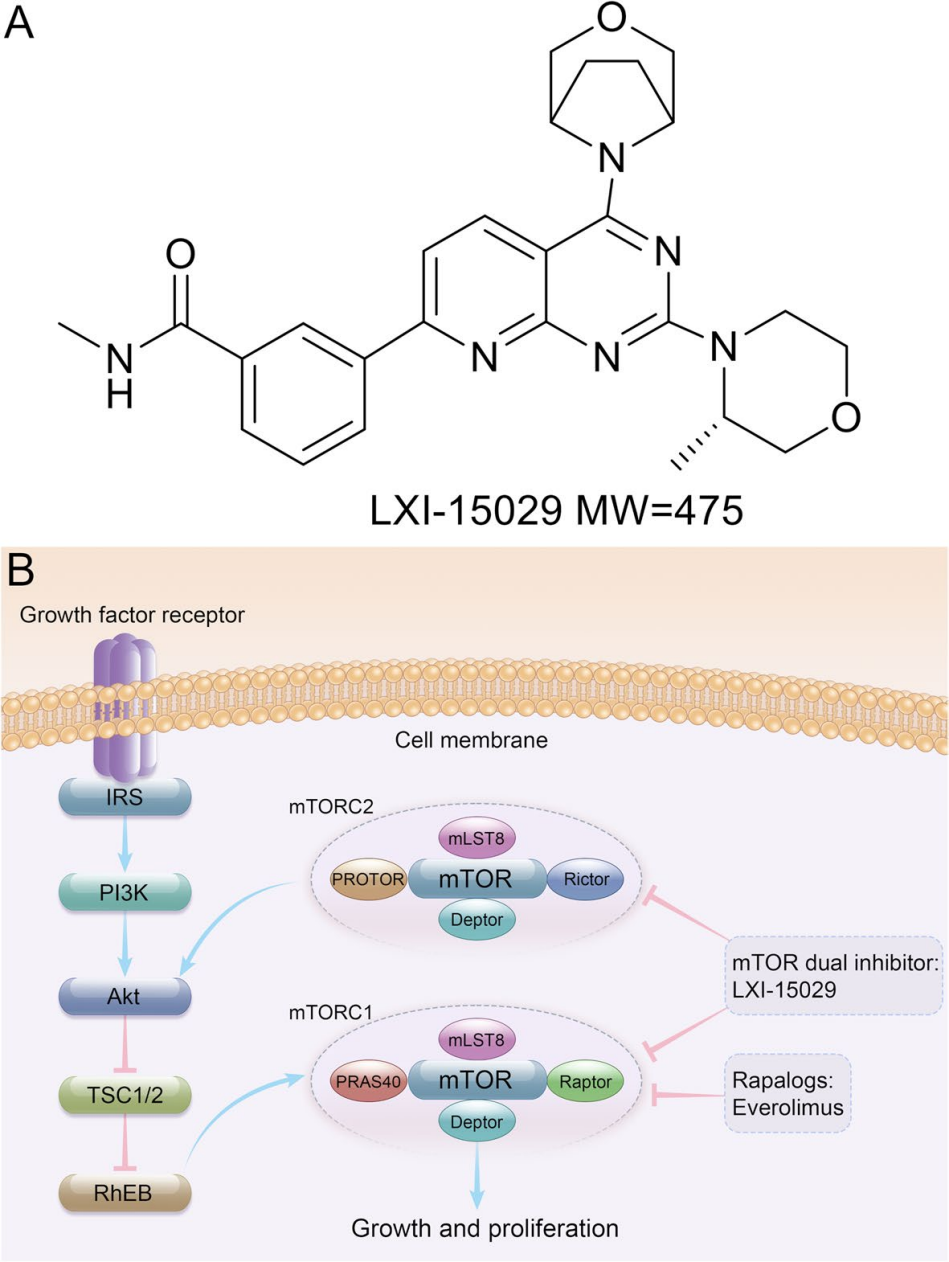
**A** Molecular structure of LXI-15029. **B** Mechanism of dual mTOR inhibitors

In order to overcome the disadvantages of first-generation mTOR inhibitors, second-generation competitive inhibitors have been developed. Various new compounds have been developed by targeting mTORC1 or mTORC2 to develop dual mTOR inhibitors [19]. Various candidates (OSI-027, TAK228, AZD8055, AZD2014, and CC-223) entered clinical trials as second-generation mTOR inhibitors [20–24]. LXI-15029 (MTI-31), an ATP-competitive mTOR kinase inhibitor with high activity and selectivity, inhibits the phosphorylation of mTOR1 substrate S6K1 and eukaryotic translation initiation factor 4E binding protein 1 (4E-BP1), mainly through the direct binding with the catalytic domain of mTOR kinase, preventing the phosphorylation of the mTOR2 substrate AKT and inhibiting the feedback activation of AKT [25–28]. LXI-15029 is a dual inhibitor for both mTORC1 and mTORC2 kinases and could theoretically be used to treat various solid tumors (Fig. 1).

The preclinical in vitro pharmacodynamic study of LXI-15029 showed a very high selectivity in inhibiting mTOR and effective inhibition of the phosphorylation of mTOR kinase substrates. LXI-15029 can bind to mTOR proteins potently, significantly, or completely inhibiting the phosphorylation of the mTORC1 substrates S6K1 T389, S6 S235/236, and 4E-BP1 T70, as well as the phosphorylation of the mTORC2 substrate AKT S473, thereby inhibiting the growth of tumor cells. In vivo pharmacodynamic studies showed that LXI-15029 had a selective antitumor activity in human renal carcinoma, gastric cancer, breast cancer, and lung cancer with abnormal regulation in the mTOR signaling pathway in nude mouse xenograft models [25, 26].

Previous preclinical studies demonstrated that LXI-15029, the mTORC1/2 dual inhibitor, showed significant selectivity and higher inhibitory effects on phosphorylation of substrates by mTOR kinases, compared with rapalogs in preclinical studies [25, 26], but no trials are available regarding the effects of LXI-15029 in patients with metastatic solid tumors. Therefore, this study aimed to evaluate the safety, tolerability, pharmacokinetic (PK) profile, and preliminary efficacy of LXI-15029 in Chinese patients with metastatic malignant solid tumors.

## Methods

### Patient eligibility

Patients were eligible for enrollment based on the following criteria: 1) 18 to 65 years of age; 2) histologically or cytologically diagnosed with advanced malignant solid tumors; 3) failure of routine therapy or without conventional standard therapy; 4) Eastern Cooperative Oncology Group Performance Status (ECOG PS) 0–1; 5) life expectancy of > 3 months; 6) adequate or acceptable organ and bone marrow function. The patients were excluded if they had received prior or current treatment with PI3K or mTOR inhibitors.

### Study design

This was a single-center, open-label, phase I study to determine the safety, PK, the maximum tolerated dose (MTD), and preliminary efficacy of LXI-15029 monotherapy in Chinese patients with advanced malignant solid tumors. LXI-15029 capsules (provided by Shandong Luoxin Pharmaceutical Group Co., Ltd.) were administered orally twice daily (BID) under the fasting state. A conventional 3 + 3 dose escalation design was used to explore the MTD. At least three participants with evaluable dose-limiting toxicity (DLT) and tolerability were included in each dose group. According to the National Cancer Institute Common Terminology Criteria for Adverse Events version 4.0.3 (NCI-CTCAE 4.0.3), DLTs were defined as adverse events (AEs) or abnormal laboratory values unrelated to disease progression and reasonably likely to be related to the investigational product within 30 days after the first dose (cycle 1), as grade 2 non-infectious pneumonia or abnormal renal function (increased creatinine), any grade 3 non-hematological AEs, grade 3 neutropenia with fever or thrombocytopenia, or any grade 4 hematological AEs.

At least three participants were enrolled in each cohort. Participants were enrolled in the next dose cohort if no DLT was observed within 30 days after the first dose. If one case of DLT occurred, three additional participants would be enrolled in this cohort. Then, if no additional DLT occurred, participants were enrolled in the next higher-dose cohort. If additional DLTs occurred, the current dose was considered intolerable and enrollment in this cohort was discontinued. The decision to explore intermediate doses or define the previous dose level as the MTD was made by the Safety Review Committee (SRC). Participants with unevaluable DLT were replaced by another participant with evaluable DLT.

The starting dose of LXI-15029 as a non-cytotoxic antitumor drug in this first-in-human phase I clinical trial was calculated as ≥ 1/5 of the no observed adverse effect level (NOAEL) in non-rodent animals, obtained in preclinical trials. The participants received LXI-15029 monotherapy in each dose cohort and multiple doses of LXI-15029 for 4 weeks (cycle 1) following a 1-day washout period after a single dose of LXI-15029. The participants continued therapy with LXI-15029 if good safety and tolerability were assessed by the investigators after one cycle of treatment. Five ascending dose groups of LXI-15029 BID (10, 20, 40, 60, and 80 mg) were planned to be evaluated, with 3 to 6 evaluable patients who received LXI-15029 monotherapy in each cohort. The following cohort was given dose escalation until the intolerable dose. The investigators could select higher doses (i.e., 110 and 150 mg, with reference to the modified Fibonacci method) if the 80 mg BID dose was well tolerated. The participants continued therapy with LXI-15029 until progressive disease (PD), the occurrence of intolerable toxicity, or withdrawal of consent.

All interventions in this study were conducted in accordance with the Declaration of Helsinki guidelines of the International Conference for Harmonization/Good Clinical Practice guidelines and approved by the Independent Ethics Committee (IEC) of the National Cancer Center/Cancer Hospitals. Written informed consent was obtained from all study participants before enrollment. The first registration at ClinicalTrial.gov is 24/04/2017 with full registration number NCT03125746.

### Endpoints

The primary endpoint was the safety and tolerability of LXI-15029 monotherapy, including confirmation of MTD. The secondary endpoints were the PK parameters and investigator-assessed antitumor effect, including objective response rate (ORR, defined as the proportion of patients with complete response [CR] and partial response [PR] as the best overall response), disease control rate (DCR, defined as the proportion of patients with CR, PR or stable disease [SD] ≥ 6 weeks) and progression-free survival (PFS) according to the Response Evaluation Criteria in Solid Tumors (RECIST) version 1.1.

### PK analysis

Blood samples for PK analysis of LXI-15029 were collected on day 1 (pre-dose, 20 min, 40 min, 1 h, 1.5 h, 2 h, 3 h, 4 h, 6 h, 8 h, 12 h, 24 h, and 48 h), cycle 1 day 8 (pre-dose), cycle 1 day 15 (pre-dose), and cycle 1 day 28 (pre-dose, 20 min, 40 min, 1 h, 1.5 h, 2 h, 3 h, 4 h, 6 h, 8 h, and 12 h). The blood samples were stored at -70°C until testing. The concentration of LXI-15029 in plasma was measured using the validated liquid chromatography-tandem mass spectrometry (LC–MS/MS) method. The concentration–time information of LXI-15029 was summarized based on pre-dose and post-dose data of each patient, to explore the linearity of exposure with single dose and steady-state situations, the time to reach steady state and the predictability of single to multiple doses PK. PK parameters were calculated separately for single and repeated doses using descriptive statistics. PK analysis was performed by Covance Central Laboratory using the validated software Phoenix WinNonlin Version 8.1 (Pharsight, Mountain View, CA). A standard non-compartmental method was used to calculate the peak plasma concentration (C_max_), time to reach the C_max_ (t_max_), the total area under the concentration–time curve (AUC_0-∞_), and the elimination half-life (t_1/2_).

### Patient assessments and evaluation

All patients who received at least one dose of LXI-15029 were included in the safety and toxicity evaluations. Safety evaluations were performed on days 1, 8, 15, and 22 in cycle 1 and once every month after that. The safety assessments included vital signs, laboratory tests, and an electrocardiogram. Safety data were evaluated separately. At the end of the study, all safety data were analyzed appropriately according to the statistical analysis plan. The AEs were coded using the Medical Dictionary for Regulatory Activities (MedDRA version 24.0). The AE severity was graded according to the NCI-CTCAE 4.03.

The antitumor effects of LXI-15029 monotherapy was evaluated based on ORR, DCR, and PFS according to RECIST version 1.1 on week 4 and every 8 weeks thereafter, and the best overall response was confirmed at least 4 weeks after the initial response.

### Statistical analysis

All safety, tolerance, PK, and antitumor data from each dose group were tabulated and summarized according to the statistical analysis plan. The statistical analysis of the study was descriptive. The continuous variables were described as mean ± standard deviation or median (minimum, maximum). For the PK data, geometric mean and geometric mean coefficient of variation were also provided. The categorical variables were described as n (%). PFS was summarized using the Kaplan–Meier method. Percentiles (25%, median, 75%) of the event time distribution were presented along with their two-sided 95% confidence interval (CI). All data processing, summaries, and analyses were performed using SAS 9.4 (SAS Institute, Cary, NC, USA).

## Results

### Characteristics of the participants

**Fig. 2:**
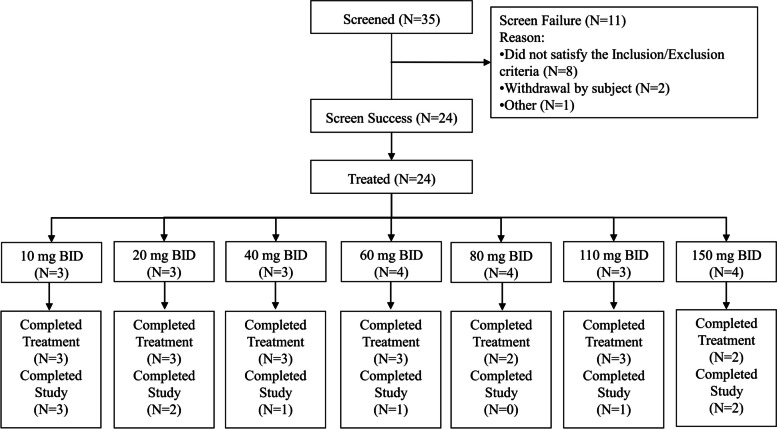
Flowchart of patient disposition

Between June 2017 and July 2021, 35 patients were screened at the National Cancer Center China, of which 11 (31.4%) patients were screen failures, and 24 (68.6%) participants were successfully enrolled (Fig. 2). At the time of study entry, all participants were diagnosed with advanced malignant solid tumors confirmed histologically or cytologically. Nineteen participants completed treatment, and five participants discontinued treatment. The reasons for discontinuation included 2 (8.3%) cases of AEs, 1 (4.2%) of consent withdrawal, 1 (4.2%) of protocol deviation, and 1 (4.2%) of DLT. The baseline characteristics of the participants are summarized in Table 1.

**Table 1:** Demographic and baseline characteristics

**Characteristics**	Median (range)/n (%)
Age (years)	47.5 (24–66)
Sex	
Male	8 (33.3)
Female	16 (66.7)
ECOG	
0	4 (16.7)
1	20 (83.3)
Tumor type	
Breast cancer	10 (41.7)
Fibrosarcoma	4 (16.7)
Leiomyosarcoma	4 (16.7)
Adrenal gland cancer	1 (4.2)
Chondrosarcoma	1 (4.2)
Neuroendocrine carcinoma	1 (4.2)
Neurofibrosarcoma	1 (4.2)
Soft tissue sarcoma	1 (4.2)
Synovial sarcoma	1 (4.2)
Prior systemic therapies	
1–2	9 (37.5)
3–5	11 (45.8)
> 5	4 (16.7)

*ECOG* Eastern Cooperative Oncology Group

### Safety

LXI-15029 orally administered BID was well tolerated at all dose levels from 10 to 150 mg. Only one participant at 150 mg BID experienced a DLT within the DLT observation window. The DLT event was a grade 3 increased alanine aminotransferase (ALT), which recovered after symptomatic supportive therapy. Since only three participants were enrolled in the 150 mg cohort and one had a DLT, the MTD of LXI-15029 monotherapy in Chinese patients with advanced solid tumors was 110 mg BID.

All 24 participants had treatment-emergent AEs (TEAEs), of which 22 had treatment-related AEs (TRAEs). Five participants had serious TEAEs, of which one had a serious TRAE. Three participants had TEAEs leading to LXI-15029 discontinuation, of which one had a TRAE. Eleven patients had TEAEs occurring within 30 days after LXI-15029 discontinuation.

Nearly all TRAEs were CTCAE grade 1 (62.5%) and grade 2 (25%), except the participant with a DLT. The most common TRAEs were leukocytopenia (41.7%), increased ALT (20.8%), increased aspartate aminotransferase (AST) (20.8%), prolonged QT interval (20.8%), and hypertriglyceridemia (20.8%). The TEAEs that occurred in the escalation dose groups are shown in Table 2.

**Table 2:** Summary of TEAEs by PT and maximum CTCAE grade

Preferred term, n (%)	10 mg BID (*n* = 3)	20 mg BID (*n* = 3)	40 mg BID (*n* = 3)	60 mg BID (*n* = 4)	80 mg BID (*n* = 4)	110 mg BID (*n* = 3)	150 mg BID (*n* = 4)	Total (*n* = 24)
Patients with any TEAE	3 (100.0)	3 (100.0)	2 (66.7)	3 (75.0)	4 (100.0)	3 (100.0)	4 (100.0)	22 (91.7)
Grade 1	3 (100.0)	2 (66.7)	1 (33.3)	1 (25.0)	3 (75.0)	3 (100.0)	2 (50.0)	15 (62.5)
Grade 2	0	1 (33.3)	1 (33.3)	2 (50.0)	1 (25.0)	0	1 (25.0)	6 (25.0)
Grade 3	0	0	0	0	0	0	1 (25.0)	1 (4.2)
≥ 10% TRAE								
Leukocytopenia	0	1 (33.3)	2 (66.7)	1 (25.0)	2 (50.0)	1 (33.3)	3 (75.0)	10 (41.7)
ALT increased	0	1 (33.3)	0	1 (25.0)	0	1 (33.3)	2 (50.0)	5 (20.8)
AST increased	0	0	0	1 (25.0)	0	2 (66.7)	2 (50.0)	5 (20.8)
ECG QT prolonged	0	0	0	2 (50.0)	1 (25.0)	2 (66.7)	0	5 (20.8)
Hypertriglyceridemia	0	1 (33.3)	1 (33.3)	2 (50.0)	0	0	1 (25.0)	5 (20.8)
Sinus bradycardia	0	1 (33.3)	0	1 (25.0)	1 (25.0)	0	1 (25.0)	4 (16.7)
Diarrhea	0	0	0	2 (50.0)	2 (50.0)	0	0	4 (16.7)
Fatigue	0	1 (33.3)	0	1 (25.0)	2 (50.0)	0	0	4 (16.7)
GGT increased	0	1 (33.3)	0	1 (25.0)	0	0	2 (50.0)	4 (16.7)
Neutropenia	0	0	0	1 (25.0)	0	0	3 (75.0)	4 (16.7)
LDL increased	0	1 (33.3)	1 (33.3)	1 (25.0)	0	0	0	3 (12.5)
Decreased appetite	1 (33.3)	0	0	0	1 (25.0)	0	1 (25.0)	3 (12.5)
Hypercholesterolemia	0	0	0	3 (75.0)	0	0	0	3 (12.5)

*BID* twice daily, *TEAEs* treatment-emergent adverse events, *TRAE* treatment-related adverse event, *PT* preferred term, *CTCAE* Common Terminology Criteria for Adverse Events, *ALT* alanine aminotransferase, *AST* aspartate aminotransferase, *ECG* electrocardiogram, *GGT* γ-glutamyl transferase, *LDL* low-density lipoprotein

No apparent associations of AE rates, laboratory test parameters, vital signs, ECG, physical examination, or other safety data and dose levels were observed based on the descriptive results. The mean ALT increased slightly from baseline except at the 10-mg and 80-mg doses. The mean AST increased slightly from baseline except at the 10-mg, 60-mg, and 80-mg doses.

One patient died within 28 days of the last dose of LXI-15029, which was attributed to disease progression unrelated to treatment.

### PK

**Fig. 3:**
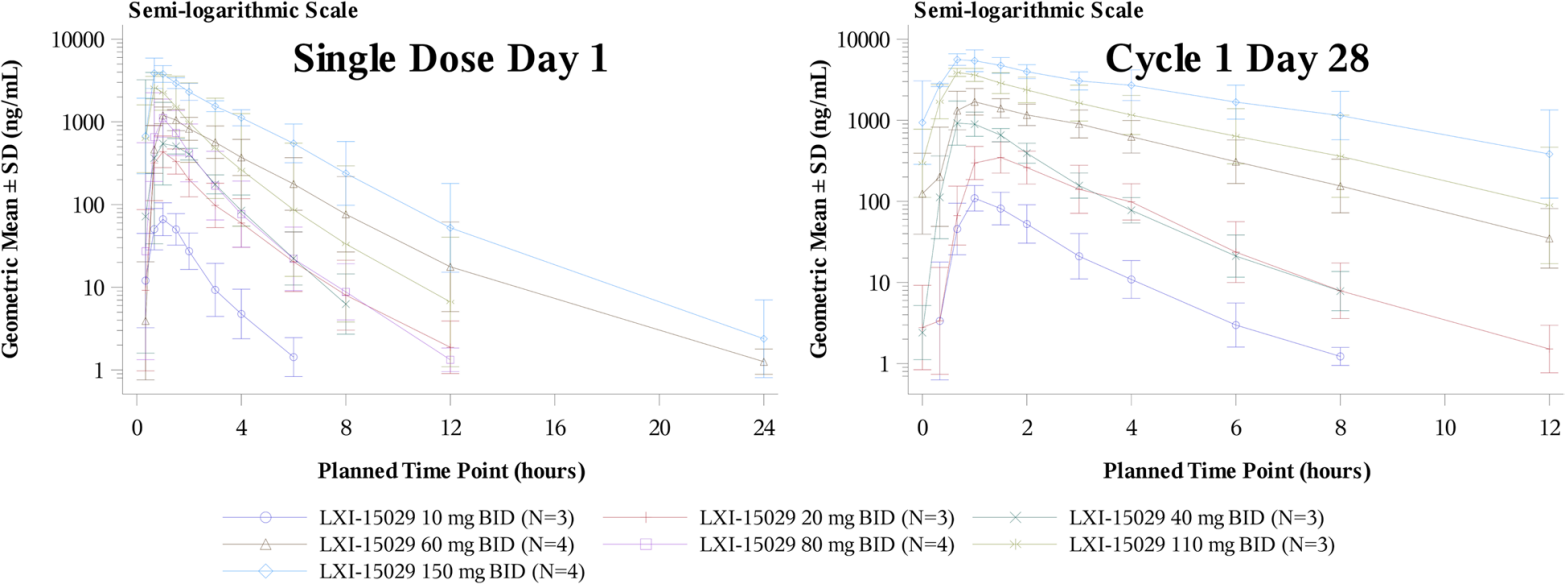
Arithmetic mean ± standard deviation of plasma concentrations on single dose day 1 and cycle 1 day 28

The blood samples from all 24 participants were collected for PK analysis. LXI-15029 was fast absorbed following oral administration, and the median range of T_max_ was 0.68–1.25 h after a single dose on day 1. The median range of T_max ss_ was 0.67–1.47 h after dosing on cycle 1 day 28 over the dose range of 10 to 150 mg. The exposure (C_max_ and AUC) of LXI-15029 increased with the dose increase from 10 to 150 mg, except for 80 mg on day 1. On cycle 1 day 28, the C_max ss_ of LXI-15029 increased with the dose increase from 10 to 150 mg. C_min ss_, C_av ss_, and AUC_ss_ of LXI-15029 increased with the dose increase from 10 to 150 mg, except for 80 mg. For C_max_ and AUC evaluations, the increase of C_max_ and AUC was greater than dose proportionality over the dose range on single dose day 1 and cycle 1 day 28. The values of the PK parameters are shown in Tables 3, 4 and Fig. 3.

**Table 3:** Summary of single-dose pharmacokinetic (PK) parameters of LXI-15029 (cycle 1 day 1)

Parameter	10 mg BID (*n* = 3)	20 mg BID (*n* = 3)	40 mg BID (*n* = 3)	60 mg BID (*n* = 4)	80 mg BID (*n* = 4)	110 mg BID (*n* = 3)	150 mg BID (*n* = 4)
C_max_ (ng/mL)	66.47 (48.24)	473.90 (64.20)	1007.50 (70.54)	1338.72 (15.09)	1336.64 (51.33)	2934.68 (30.12)	4140.93 (35.35)
AUC_0-t_ (h*ng/mL)	108.41 (62.90)	890.76 (51.53)	1675.07 (32.42)	3728.54 (27.79)	1861.40 (65.58)	5333.37 (78.45)	12029.13 (17.03)
AUC_0-T_ (h*ng/mL)	110.86 (60.64)	893.53 (50.94)	1678.07 (32.52)	3667.10 (27.47)	1868.12 (65.15)	5313.27 (77.58)	11772.83 (15.46)
AUC_0-∞_ (h*ng/mL)	110.96 (60.64)	897.84 (51.09)	1682.11 (32.15)	3750.67 (28.07)	1870.62 (65.13)	5341.42 (78.38)	12059.73 (17.01)
T_max_ (h)	0.98 (1.0, 1.0)	1.00 (0.7, 1.0)	0.68 (0.7, 2.0)	1.25 (1.0, 1.5)	0.87 (0.7, 1.5)	0.70 (0.7, 1.0)	0.82 (0.7, 1.0)
t_1/2_ (h)	0.95 ± 0.205	1.47 ± 0.268	1.13 ± 0.324	2.00 ± 0.599	1.33 ± 0.174	1.48 ± 0.531	1.94 ± 0.706
V/F (L)	126.07 ± 40.383	49.56 ± 19.453	42.35 ± 25.602	45.88 ± 14.063	92.88 ± 47.995	43.99 ± 17.319	34.75 ± 12.576
CL/F (L/h)	99.45 ± 51.035	24.11 ± 11.940	24.58 ± 7.772	16.47 ± 4.677	48.30 ± 25.396	24.35 ± 17.791	12.57 ± 2.022

Median (min, max) is presented for T_max_. The arithmetic mean ± standard deviation is presented for t_1/2_, V/F, and CL/F. The geometric mean (coefficient of variation) is presented for the other PK parameters

*BID* twice daily, *C*_*max*_ peak concentration, *AUC* area under the curve, *T*_*max*_ time to reach C_max_, t_1/2_: half-life, *V/F* apparent volume of distribution, *CL/F* apparent clearance

**Table 4:** Summary of multiple-dose pharmacokinetic (PK) parameters of LXI-15029 (cycle 1 day 28)

Parameter	10 mg BID (*n* = 3)	20 mg BID (*n* = 3)	40 mg BID (*n* = 3)	60 mg BID (*n* = 4)	80 mg BID (*n* = 4)	110 mg BID (*n* = 3)	150 mg BID (*n* = 4)
C_max ss_ (ng/mL)	114.53 (33.59)	350.08 (46.00)	1013.97 (49.26)	1689.82 (38.99)	2260.53 (76.33)	3929.34 (10.26)	5838.12 (21.53)
C_min ss_ (ng/mL)	-	1.61 (91.19)	3.74 (12.32)	35.01 (102.00)	6.41 (41.80)	152.19 (924.66)	452.06 (256.69)
C_av ss_ (ng/mL)	15.89 (32.34)	71.93 (53.96)	139.30 (32.61)	477.10 (48.87)	434.73 (63.85)	1075.06 (41.95)	2121.25 (34.81)
AUC_ss_ (h*ng/mL)	190.63 (32.34)	863.16 (53.96)	1671.61 (32.61)	5725.21 (48.87)	5216.71 (63.85)	12900.71 (41.95)	25454.95 (34.81)
T_max ss_ (h)	1.02 (1.0, 1.6)	1.47 (1.0, 1.5)	0.67 (0.7, 1.0)	0.98 (1.0, 1.0)	1.08 (0.7, 1.5)	0.67 (0.7, 1.0)	0.98 (0.7, 1.0)
t_1/2ss_ (h)	1.13 ± 0.080	1.24 ± 0.167	1.14 ± 0.250	1.90 ± 0.197	1.33 ± 0.068	2.33 ± 0.857	3.26 ± 1.247
Rac_Cmax_	1.96 ± 1.061	0.75 ± 0.140	1.02 ± 0.180	1.37 ± 0.382	1.28 ± 0.102	1.37 ± 0.352	1.36 ± 0.507
Rac_AUC_	2.08 ± 1.289	0.99 ± 0.260	1.00 ± 0.036	1.47 ± 0.546	1.78 ± 0.268	2.51 ± 0.778	2.39 ± 0.768

Median (min, max) is presented for T_max ss_. The arithmetic mean ± standard deviation is presented for t_1/2ss_, Rac_Cmax_, and Rac_AUC_. The geometric mean (coefficient of variation) is presented for the other PK parameters

*BID* twice daily, *C*_*max ss*_ steady-state peak concentration, *C*_*min ss*_ steady-state trough concentration, *C*_*av ss*_ the average value of the steady-state concentration, *AUC* area under the curve, *T*_*max*_ time to reach C_max ss_, *t*_*1/2*_ half-life, *Rac* accumulation ratio

### Preliminary efficacy data

Of the 24 participants who completed the treatment plan, 20 were evaluated for therapeutic efficacy. The tumor target lesions ranged from one to five. The lung, liver, and lymph nodes were the most common target lesion sites. Efficacy was evaluated based on the oncological evaluation analysis set and investigator assessment as per RECIST 1.1. No participants achieved CR or PR. The best overall response was assessed as SD, and the DCR was 40% (8/20; 95% CI 21.7–60.6). No DCR-dose correlation was observed. One participant with fibrosarcoma received 20 mg BID LXI-15029 and had SD at cycles 1 to 11 and then PD at cycle 12; the duration of SD was 344 days.

**Fig. 4:**
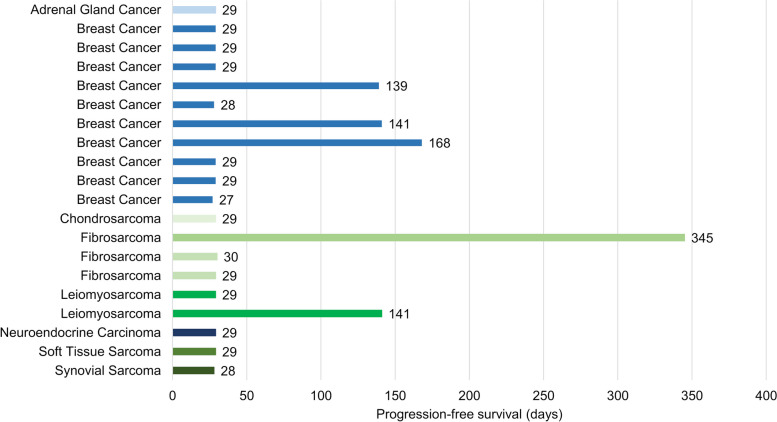
Progression-free survival of 20 evaluable participants

Survival analysis showed that the overall median PFS of the participants from the date of the first dose of LXI-15029 was 29 days (95% CI: 29–141) (Fig. 4). The median PFS was 0.95 months (95% CI: 0.89–4.63) in patients with breast cancer and 0.97 months (95% CI: 0.92–11.33) in patients with sarcoma.

## Discussion

The discovery of mTOR represents a fundamental breakthrough in the understanding of cell growth, metabolism, and disease [1]. Understanding the critical role of the mTOR pathway in tumor growth has driven a growing number of PI3K/AKT/mTOR inhibitors that have not yet achieved significant clinical success. The present open-label first-in-human, single and multiple ascending doses phase I study in Chinese patients with advanced malignant solid tumors evaluated the tolerability, PK profiles, and preliminary efficacy of LXI-15029, a novel mTORC1/2 dual inhibitor. LXI-15029 was well tolerable when administered orally over the dose range of 10 to 150 mg BID. In the escalation dose groups, TRAEs were mostly mild or moderate. Only one DLT was observed in the 150-mg BID group, as determined by the investigator, andthe remaining participants could tolerate the treatment without any serious TRAEs.

The serious toxicities of PI3K/AKT/mTOR inhibitors limit their clinical use and approval, including hyperglycemia, hyperlipidemia, bone marrow suppression, pneumonia, stomatitis, and hepatotoxicity [16]. In the present trial, the most common AEs were hematological toxicities (leukocytopenia), abnormal liver function (elevated ALT and AST), prolonged QT interval, and hypertriglyceridemia. The AE pattern differs from those observed in previous clinical trials for other mTOR inhibitors, in which the most common AEs were stomatitis, anorexia, anemia, dyspnea, and pneumonitis [10, 29]. Interestingly, the AE spectrum observed in the present trial was similar to that observed with everolimus in Chinese patients with hormonal-positive advanced breast cancer, such as the MIRACLE trial [9, 30]. These distinct AE patterns could be attributable to the genetic characteristics of the different ethnic groups. Of note, the safety of LXI-15029 seems to be better than everolimus plus exemestane in the BOLERO-5 trial [31] and everolimus plus letrozole in the MIRACLE trial [9], which were also conducted in Chinese females with advanced breast cancer. Indeed, in the BOLERO-5 trial, 45% of the participants had grade ≥ 3 AEs. In the MIRACLE trial, grade ≥ 3 stomatitis and hypertriglyceridemia occurred in 9.9% and 12.9% of the participants, respectively. In the present study, only one DLT (grade 3 increased ALT) occurred with LXI-15029 150 mg BID, and all other TRAEs were grade 1–2. The level of toxicity appears to be acceptable for this patient population. Therefore, LXI-15029 might have the potential to be used in combination with other drugs. There is a need to analyze the mechanisms leading to mTOR inhibitors toxicity, which might help develop optimal prevention and treatment strategies.

The PK profile showed that LXI-15029 was absorbed rapidly, and the arithmetic mean of t_1/2_ was 0.95–2.00 h after single dosing and 1.13–3.26 h after multiple dosings across all dose cohorts. For C_max_ and AUC evaluations, the increases in C_max_ and AUC were greater than the dose proportionality over the 10-mg to 150-mg single and repeated doses on day 1 and cycle 1 day 28. The in vivo preclinical data showed that LXI-15029 had a selective antitumor effect, its activity was dose-dependent in the dose range of 5–40 mg/kg, and the onset dose varied to a certain degree in different tumor models. Compared with the preclinical data, the exposure of LXI-15029 in the 150-mg BID group was far higher than the effective dose in preclinical models and threefold higher than the highest non-severely toxic dose (HNSTD) found in rats and fivefold higher than the NOAEL in dogs. Considering that one DLT event was reported in the 150-mg BID group, which was nearly twice the predefined maximum dose of 80 mg BID in the initial protocol, and the limited benefits for the patients in high-dose groups, dose escalation was terminated after three participants were enrolled in the 150-mg BID cohort. According to the exposure-efficacy analysis, no relationship was found between C_trough_ and median PFS or DCR. Similarly, the LXI-15029 C_trough_ level did not significantly predict long-term efficacy in the present study. These findings suggest that higher LXI-15029 exposure would not translate into survival benefits, probably because of decreased tolerability due to toxicities such as severe stomatitis and anorexia.

Many studies aim to understand the potential resistance mechanisms of mTOR-targeted therapies, which is necessary for the rational application of mTOR inhibitors for the effective treatment of cancer. mTOR inhibitors interrupt the phosphorylation of substrates and block relevant signal transduction, thereby inhibiting the cell cycle, tumor metabolism, and cell survival and exerting antitumor effects through binding with the mTOR binding site [32]. Previous studies showed that monotherapy with a mTORC1/2 dual inhibitor might have no superior efficacy than the mTORC1 inhibitor everolimus [33]. Still, incomplete inhibition of mTORC1/2 can lead to compensatory activation of different pathways and inhibition of negative feedback loops [17, 18]. The two mTOR complexes, mTORC1 and mTORC2, have different sensitivities to rapamycin. These properties of rapamycin are mainly attributed to the competition between rapamycin and phosphatidic acid for mTOR [34, 35]. mTOR inhibitors might also be combined with endocrine therapy and could help regulate the PI3K/AKT/mTOR pathway and reverse the resistance to CDK4/6 inhibitors [36, 37]. Combination therapies based on mTOR inhibitors might be a key to prolonging treatment response. In addition, patients resistant to drugs targeting the extracellular pathway to the PI3K-AKT-mTOR pathway often display an overactivation of the mTOR signal [38], and these patients might benefit from dual mTORC1/2 inhibition.

Cancer immunotherapy is another promising approach for mTOR dual blockers. Cell-based and in vivo research showed that mTOR inhibitors can enhance the efficacy of various tumor immunotherapy methods by elevating PD-L1 expression in tumor cells and inhibiting the activation of T memory cells [39]. A recent case report in late-stage ovarian cancer suggests the synergistic effect of the combination of everolimus and an anti-PD-1 agent [40]. Antibody–drug conjugates (ADCs) are also relatively recent agents with promising responses and benefits, and combining ADCs with PI3K/mTOR inhibitors also appears promising. For instance, 5T4-ADC in combination with PI3K/mTOR inhibitors achieved enhanced anti-tumor activity, and the synergistic effect might be attributed to the mechanism of action of payload [41]. Furthermore, overexpression of the PI3K/AKT/mTOR pathway was related to acquired resistance to gemtuzumab ozogamicin in acute myelocytic leukemia cells, and PI3K/AKT/mTOR inhibitors could lead to re-sensitization of the resistant cells [42].

Notably, about 50% of the participants had rare cancers such as sarcoma and neuroendocrine carcinoma, which have achieved few treatment breakthroughs. The results in this study suggest favorable efficacy for these rare cancers. Still, patients who are more likely to benefit from mTOR inhibitors remain to be further explored. Patients resistant to targeted therapy such as epidermal growth factor receptor tyrosine kinases inhibitors might be potential beneficiaries since they often have activation of the PI3K/AKT/mTOR pathway. Moreover, some ongoing clinical trials could suggest that mTOR dual inhibition might be beneficial in patients harboring NFE 2L2, STK11, RICTOR, or other specific genetic alterations (NCT04518137) or combined with anti-PD-1 antibody (NCT04337463) or antineoplastic exportin-1 (XPO1) inhibitors (NCT04998760) [43]. Further research is required to focus on targetable sites of mTOR and potential drug targets for destabilization of the mTOR complex and to understand the extent of efficacy from optimal combination regimens of mTOR inhibitors and other therapies [44–47]. The excellent safety profile of LXI-15029 demonstrated in the present study might provide more opportunities to evaluate the efficacy in the setting of combination therapy.

The limitations of this study included the small sample size, the single-center design, and the lack of tissue and serum collection before and during the study. Collection of tissue and serum samples in future studies would allow exploring predictive biomarkers and facilitate observing the changes in the PI3K/AKT/mTOR signaling pathway and related specific genetic alterations, which will further improve treatment strategies and guide the better selection of responsive patients [48]. Despite these limitations, the present study suggests that increased exposure to LXI-15029 is associated with an increased risk of hepatic toxicity.

## Conclusions

LXI-15029 monotherapy demonstrated a favorable safety profile, tolerability, and preliminary antitumor activity in Chinese patients with advanced malignant solid tumors. PK-guided dosing might improve the efficacy and safety of LXI-15029, leading to further investigation in a larger population-based phase II study and evaluation of an effective combination with immunotherapy or other target therapy.

## Data Availability

The datasets used and/or analyzed during the present study are available from the corresponding author upon reasonable request.

## Abbreviations

ADCs: Antibody–drug conjugates
AEs: Adverse events
ALT: Aminotransferase
AST: Aspartate aminotransferase
CI: Confidence interval
DCR: Disease control rate
DLT: Dose-limiting toxicity
ECOG PS: Eastern Cooperative Oncology Group Performance Status
eIF4E: Eukaryotic initiation factor 4E
HIF: Hypoxia-inducible factor
HNSTD: Highest non-severely toxic dose
IEC: Independent Ethics Committee
LKB1: Liver kinase B1
LC–MS/MS: Liquid chromatography-tandem mass spectrometry
MTD: Maximum tolerated dose
mTOR: Mammalian target of rapamycin
MedDRA: Medical Dictionary for Regulatory Activities
NOAEL: No observed adverse effect level
ORR: Objective response rate
PK: Pharmacokinetics
PKB: Protein kinase B
PI3K: Phosphatidylinositol 3-kinase
PTEN: Phosphatase and tensin homolog
PD: Progressive disease
PFS: Progression-free survival
RECIST: Response Evaluation Criteria in Solid Tumors
SRC: Safety Review Committee
S6K1: S6 kinase 1
TSC1/2: Tuberous sclerosis complex 1/2
VEGF: Vascular endothelial growth factor
